# I Can See Clearly Now: Episodic Future Thinking and Imaginability in Perceptions of Climate-Related Risk Events

**DOI:** 10.3389/fpsyg.2020.00218

**Published:** 2020-02-21

**Authors:** Simen Bø, Katharina Wolff

**Affiliations:** Department of Psychosocial Science, Faculty of Psychology, University of Bergen, Bergen, Norway

**Keywords:** episodic future thinking, episodic foresight, future thinking, risk perception, climate risk, perceived risk, climate, open science

## Abstract

Climate change is statistical, abstract and difficult to comprehend directly. Imagining a specific, personal episode where you experience consequences of climate change in the future (episodic future thinking) may bring climate change closer, thus increasing the perceived risk of climate-related risk events. We conducted an experiment to test whether episodic future thinking increased the perceived risk of climate-related risk events and climate change in general, as compared to thinking about the future in a general, abstract manner (semantic future thinking). We also tested whether this effect is moderated by how easy it is to imagine the specific climate-related risk event initially. Participants were randomly assigned to an episodic future thinking-condition or a semantic future thinking-condition, and two of the risk events in each condition were related to flooding (difficult to imagine) and two were related to extreme temperature (easy to imagine). The results show no main effect of episodic future thinking on perceived risk, and no interaction effect with imaginability. Contrary to expectations and earlier research, this suggests that episodic future thinking may not influence risk perception.

## Introduction

Climate change threatens humans. Recent reports suggest that consequences of climate change, such as an increase in the number of floods or in days with extreme temperature, can result in widespread humanitarian catastrophe (IPCC, 2019). Because of the gravity of this threat, we need a better understanding of which factors influence climate change perceptions. Understanding the antecedents of how people perceive the risk of both specific climate-related risk events and climate change in general, and which factors predict human engagement in climate change-related issues, is of paramount importance in current environmental psychology (Stern, 2011; Gifford, 2014; Otto et al., 2014; van der Linden, 2015).

Climate change differs from other hazards. Firstly, climate change occurs over an extensive period of time, making it impossible to directly perceive the changes as they occur (Weber, 2006, 2016; van der Linden, 2015). Second, climate change is global, differing from other regional or local risks, such as terrorism or war. Third, climate change is perceived as psychologically distant; people think climate change will harm people other than themselves in the far future (Carmi and Kimhi, 2015; McDonald et al., 2015; Weber, 2016). These attributes hamper human engagement and imply that interventions which serve to bring climate change closer and make it more concrete may be fruitful to explore in empirical research.

Engagement with climate change depends on the perceived risk of climate change. *Perceived risk* refers to the product of an uncertainty aspect, i.e., how probable a negative outcome is perceived, and a severity aspect, i.e., the magnitude of the consequences of the hazard should they manifest (Brun, 1994; Wolff and Larsen, 2017). Some studies suggest that people tend to show *probability neglect* when judging perceived risk, relying almost solely on severity and disregarding probability (Sunstein, 2002; Slovic and Peters, 2006). For example, a person who imagines being aboard an air plane amidst an ongoing emergency would focus more on dying (severity) than the probability of a plane crash occurring (about the same as being hit by an asteroid; Schilling, 1999).

An important segment of risk perception research has involved exploring the psychological precursors to individual risk perception. An example of this is research on the effect of present beliefs on recollected risk estimates, suggesting that people remember the world as safer than they previously judged it to be (Fischhoff et al., 2005). Additionally, several models for understanding the precursors of climate change risk perception have been proposed, including distinctions between cognitive, experiential, socio-cultural and socio-demographic aspects (van der Linden, 2015). A prerequisite for assessing potential precursors is the use of experimental studies, necessary to establish whether the precursors have causal impacts on risk perception.

A potential precursor to risk perception which has been explored in prior research is *episodic future thinking* (EFT; Lee et al., 2018; Bø and Wolff, 2019). EFT is a form of future-oriented thinking which involves imagining specific episodes that may occur in the future (Atance and O’Neill, 2001). EFT is considered a future-oriented parallel to episodic memory: just as episodic memory is necessary to access specific, personal memories, EFT is necessary to imagine specific, personal episodes that may occur in the future (Atance and O’Neill, 2001; Szpunar, 2010). In taxonomies of future-oriented cognition, EFT is distinguished from *semantic future thinking* (SFT), which concerns a general, non-specific future (Abraham et al., 2008; Szpunar et al., 2014). There is, in other words, a distinct difference between contemplating a future hazard such as climate change in terms of personally experiencing climate-related risk events, and in terms of abstractly considering how such risk events may come to pass.

The research on EFT has been focused on its association with decision making, with studies suggesting that EFT helps people make long-term decisions which prioritize larger, later rewards over immediate, smaller rewards (Suddendorf and Corballis, 1997; Daniel et al., 2015; Bulley et al., 2016; Stein et al., 2018; Miloyan and McFarlane, 2019). While this literature has generally emphasized potential positive futures, imagining negative futures may impact how people comprehend adverse outcomes (Bø and Wolff, 2019). There are clear-cut reasons to argue that engaging in EFT may impact how people understand climate change as a hazard. EFT may bring climate change closer, and thus be of instrumental value to increasing climate change engagement (McDonald et al., 2015).

One theoretical framework that can help contextualize the rationale for assuming an association between EFT and risk perception is van der Linden’s model of climate change risk perception (van der Linden, 2015). He argues that one can understand climate change risk perception through four dimensions: experiential, cognitive, socio-cultural, and sociodemographic aspects. Experiential aspects encompass both affect and personal experience, so that general negative feelings toward a risk event, and personal experience with similar events, will heighten perceived risk. We suggest that EFT may impact risk perception through experiential pathways, through making climate change more vivid, personally relevant and psychologically proximal, in addition to serving as an evidentiary substitute for personal experience. Thus, there are clear theoretical reasons to assume that engaging in EFT will impact climate change risk perception (van der Linden, 2015).

One robust argument for assuming that EFT increases perceived risk is the relevance of vividness and availability for risk perception. Several studies suggest that climate change is perceived as abstract and statistical, and that a lack of vividness and availability reduces the perceived risk of climate-related risk events (van der Linden, 2015; Weber, 2016). Inasmuch as episodic future thoughts are vivid and available, and that the ease and vividness with which events come to mind matter for risk perception, engaging in EFT may increase risk perception (Slovic et al., 1981; Visschers et al., 2012). For example, vividly imagining being exposed to a flood while driving may make floods more available as a risk event, thus heightened the perceived risk of a future increase in the frequency of flooding.

A second argument is based on the importance of personal experience with extreme weather events for increasing climate change risk perception, and the assumption that simulations may have similar, vicarious effects (Weber, 2006; van der Linden, 2015). Some studies suggest that personal experience with extreme weather events (i.e., floods) increase the perceived risk of climate-related risk events and is related to willingness to act in the face of climate change (Weber, 2006, 2016; McDonald et al., 2015). It is assumed that people use personal experience of extreme weather as a proxy for climate change, owing to the abstract and statistical qualities which prevent direct experience of climate change (McDonald et al., 2015). Some have argued that simulations may have similar cognitive and behavioral consequences as personal experience, implying that simulations may have similar consequences as personal experience for climate change risk perception (Kappes and Morewedge, 2016). Hence, a person imagining a specific, personal episode that may occur may attribute this similar evidentiary value as personal experience. If so, EFT might be an impactful intervention to increase climate change engagement, because climate change occurs so slowly, and over such a long period of time, that it is inconceivable to gain sufficient personal experience to regard it as a threat directly (McDonald et al., 2015).

The third argument hinges on the notion of *psychological distance*, the personal, perceived remoteness of an event, comprising spatial distance, temporal distance, social distance and hypotheticality (Trope and Liberman, 2010; Zwickle and Wilson, 2013). Factors which serve to bring climate change closer significantly impact perceptions of climate change, and enhanced perceived temporal proximity to collective, pro-environmental goals increases the motivation for goal attainment (Bashir et al., 2014). Studies on EFT suggest that it aids in decision making by decreasing the psychological distance with which events are perceived (Daniel et al., 2015; Bulley et al., 2016; Stein et al., 2018). EFT could connect people to psychologically distant consequences of climate change, thus increasing perceived risk. For example, if climate change is perceived as spatially remote, imagining it occurring to you would increase spatial proximity; similarly, imagining climate change as occurring in one’s own personal future may make it more temporally proximal than reading reports about potential changes in 2100. Although some studies suggest that the relationship between psychological distance and climate change perceptions is more complex, there are definite reasons to assume that psychological distance may be an important mechanism (Jones et al., 2017; Ejelöv et al., 2018). Plainly, there are several credible reasons to assume an effect of EFT on the perceived risk of climate-related risk events and climate change, including vividness, availability, increased psychological proximity, and episodic thoughts being attributed similar evidentiary value as personal experience.

The prior arguments are unified in experiential pathways, but there are also cognitive pathways to climate change risk perceptions (van der Linden, 2015). These may include factors that influence perceived probability, and there are reasons to assume that EFT may influence climate change risk perception through cognitive pathways, specifically by increasing the perceived probability of an event’s occurrence. Research on future-oriented imagination suggests that outcomes which are easy to imagine are also perceived as more probable, and that imagining events multiple times makes them seem more likely to actually occur (Sherman et al., 1985; Szpunar and Schacter, 2013). Thus, both experiential and cognitive pathways are conceivable for why there would be an effect of EFT on the perceived risk of climate-related risk events and climate change in general.

Prior research which contextualizes the current study includes studies on the relevance of mental imagery for perceptions of environmental change and risk perceptions specifically (Leviston et al., 2014; Boomsma et al., 2016; Liu et al., 2019). For example, some studies suggest an association between how people imagine scenarios and how they perceive environmental challenges. In one study, researchers explored the vividness with which participants recalled a visual message related to environmental difficulties, showing an association between vividness and pro-environment goal-relevant thoughts, implying the importance of mental imagery for goal pursuit (Boomsma et al., 2016). We know of only one study which explored the effect of EFT on perceived risk for climate change (Lee et al., 2018). Lee et al. (2018) tested whether EFT heightens the perceived risk of specific environmental challenges, and whether this in turn affects pro-environmental behavior. EFT about possible future environmental challenges strengthened pro-environmental behavior, an effect mediated by how risky these challenges were perceived.

However, Lee et al. (2018) operationalized perceived risk using a measure of perceived probability, which is problematic in light of studies suggesting that people rely markedly more on severity than probability in judging risk (Sunstein, 2002; Slovic and Peters, 2006). Several authors have argued strongly for affective components in risk perception, implying that operationalizing perceived risk using a measure of perceived probability may not reflect an accurate relationship between EFT and perceived risk (van der Linden, 2015; Wilson et al., 2019). Also, the manipulation checks included in the study did not assess field perspective or *autonoetic consciousness*, which describes an awareness and knowledge of the experience of the event (Tulving, 1985). These are both defining features of EFT, making it difficult to assess whether the intended form of future thinking was precisely manipulated (Atance and O’Neill, 2001; Szpunar, 2010).

In addition to testing the effect of EFT on perceived risk, we also wished to pursue a possible explanation to findings in a previous study on EFT (Bø and Wolff, 2019). In that study, Bø and Wolff tested the effect of EFT on the perceived risk of future terror attacks, and found no difference compared to an SFT-condition, an active control condition or a passive control condition. One possible explanation was that terror attacks are highly vivid, making attempts at heightening perceived risk through EFT insufficient. If this is correct, then there may be an effect of EFT for hazards which are less vivid, such as climate change. Additionally, one could argue that the effect might be moderated by the imaginability of the hazard, or how difficult it is to imagine the hazard initially. Hence, we also tested whether an effect of EFT would interact with the imaginability of the climate-related risk event that participants were asked to imagine. Finally, we included measures of both personal and societal risk, in order to test whether the effect differed according to form of risk perception, reflecting the importance of this distinction in climate change risk perception (van der Linden, 2015). We thus tested interactions between future thinking and type of climate-related risk events for personal risk, a main effect of future thinking for both personal risk and societal risk, and a potential interaction between future thinking and the form of risk (personal vs. societal).

To summarize, we aimed to test the effect of EFT on the perceived risk of specific climate-related risk events and climate change in general. We also wished to test whether this effect was moderated by the imaginability of the hazard, specifically whether the effect was stronger for scenarios in which people initially have difficulty imagining the consequences. Additionally, we tested whether EFT interacted with the form of risk perception (personal vs. societal). To determine climate-related risk events which people perceive as easy or difficult to imagine, we first conducted a pilot study, prior to our main study, which is described below.

## Pilot Study

### Materials and Methods

#### Participant Recruitment and Questionnaire

To test which climate-related risk events people have difficulty imagining, we conducted a pilot study on tourists visiting Bergen. The pilot study was conducted as part of a larger study exploring various aspects of tourists’ mental processes and behavior. Tourists were approached in “low threshold”-places in Bergen, such as Mount Fløyen (“Fløyfjellet”). Potential respondents were approached by a research assistant and asked if they were currently on a vacation. If so, they were asked if they were willing to participate in a study by filling out a questionnaire concerning “various aspects of being a tourist.”

The questionnaire was four pages long and included various demographic questions and tourist-related questions. Only measures relevant to describing the sample and describing the current pilot study are described here. Participants were asked to answer questions pertaining to each of the following climate-related risk events: Extreme temperatures (more very warm and very cold days), increased number of floods, increased number of extinct species, changes in the arrival of seasons (i.e., spring arriving later than usual) and changes in biomes (i.e., an expansion of savannas or retreating tropical forests). Participants were randomly assigned to either answer questions with reference to themselves (personal) or with reference to others (societal). For both versions of the questionnaire, participants were asked to answer how easy they thought it was to imagine the consequences of the climate-related risk event (personal consequences vs. consequences in general), how likely it was that the climate-related risk event would happen, and how high the risk of the climate-related risk event was. All items were on a scale from 1 to 7, with endpoints very difficult/unlikely/risky and very easy/likely/risky.

#### Sample

A total of 1666 participants (929 women, *M*_*age*_ = 40.8, *SD*_*age*_ = 0.42) participated in the overall study. For our subset of the study, where participants were asked questions concerning climate change scenarios, there were 840 participants (481 women, *M*_*age*_ = 41.2, *SD*_*age*_ = 0.59).

### Results

To test whether there was a main effect of type of risk event and an interaction between version (personal vs. societal) and type of event, we conducted a two-way factorial mixed ANOVA. There was an interaction between type of event and version, Wilks’ Lambda = 0.98, *F*(1,761) = 3.75, *p* = 0.005, $\eta_{\text{p}}^{2}$ = 0.19, meaning that the difference between how easy it is to imagine the personal consequences and general consequences of the climate-related risk events differed depending on the type of event. There was also a significant main effect of type of scenario, Wilks’ Lambda = 0.94, *F*(4,761) = 12.08, *p* < 0.001, $\eta_{\text{p}}^{2}$ = 0.06, suggesting that in general, the perceived risk differed depending on type of climate-related risk event.

Because the aim of the study was to determine which climate-related risk events are the most difficult to imagine initially, and which are the easiest to imagine initially, we explored with the aim of determining for which climate-related risk event there was the greatest difference between ratings of imagining personal and general consequences, and for which climate-related risk event there was the smallest difference. The largest difference was for flooding, *t*(796) = −3.39, *p* = 0.001, *d* = −0.24: Participants asked to imagine the personal consequences of flooding (*M* = 4.30, *SD* = 1.60) had more difficulty imagining the consequences than participants asked to imagine the general consequences of flooding (*M* = 4.68, *SD* = 1.56). The smallest difference was for extreme temperature, *t*(814) = −0.21, *p* = 0.831, *d* = −0.01: Participants asked to imagine the personal consequences of extreme temperature (*M* = 4.66, *SD* = 1.57) did not have more difficulty imagining the consequences than participants asked to imagine the general consequences of extreme temperature (*M* = 4.68, *SD* = 1.58). However, we note that while there was a greater difference for floods than extreme temperature, this difference was small in size.

Concerning the relationship between ease of imagination and perceived risk, and between ease of imagination and perceived probability, there was a moderate association between ease of imagination and perceived risk (*r* = 0.45, *n* = 738, *p* < 0.001), and between ease of imagination and perceived probability (*r* = 0.44, *n* = 746, *p* < 0.001).

### Discussion

The results from our pilot study suggests that there was a clear difference in the distinction between imagining personal and general consequences; there was no difference in this distinction for extreme temperature, and a small difference in this distinction for floods. Based on these results, we argue that extreme temperature may serve as a climate-related risk event with which people initially can easily imagine personal consequences, whereas flooding may serve as a climate-related risk event with which people have difficulty initially imagining personal consequences. Thus, these scenarios are suitable for testing our assumed interaction between EFT and imaginability in perceptions of climate-related risk events for our main study.

## Materials and Methods

### Overview and Hypotheses

To test whether EFT can increase the perceived risk of climate change, we conducted a pre-registered experiment with a factorial mixed design. There were two factors: future thinking (episodic vs. semantic) and ease of imagination (easy to imagine vs. difficult to imagine). Future thinking was manipulated between subjects, and ease of imagination was manipulated within subjects. All participants were exposed to both the easy scenario and the difficult scenario, and as such, there were two conditions: (1) one EFT-condition with two easy and two difficult scenarios, and (2) one SFT-condition with two easy and two difficult scenarios. Participants were randomly assigned to EFT or SFT, and the order of the presentation of scenarios was counterbalanced.

We conducted the experiment as an online survey experiment using Qualtrics. Prior to data collection, we pre-registered the study on Open Science Framework (OSF).^1^ The data and full descriptions of both the experimental manipulation and the procedure are available in the OSF-folder for this study.^2^ Deviations from the pre-registration are reported in the remainder of the manuscript and summarized in a file in the OSF-folder.

Hypotheses for this study pertained both to the manipulation checks and the effect of future thinking on perceived risk.

(1) Participants assigned to the EFT-condition will have a higher mean score than participants assigned to the SFT-condition on the index of vividness, field perspective and autonoetic consciousness.
(2) Participants in both experimental conditions will have mean scores above the midpoint (4) on the item measuring the degree to which they thought about the future, both for the easy scenarios and for the difficult scenarios.
(3) Participants in both experimental conditions will judge the future thinking-task as more difficult for the “difficult” scenarios than for the “easy” scenarios.

Only these hypotheses concerning the effect of the independent variables on the dependent variable were tested in the confirmatory part of the analyses:

(1) There will be a main effect of future thinking on perceived risk for specific climate-related risk events, so that participants assigned to the EFT-condition will perceive the risk of specific climate-related risk events as higher than participants assigned to the SFT-condition. We predict a main effect of future thinking both for the measure of personal risk and the measure of societal risk. Statistically, this will be reflected by a significant main effect of the between-subjects factor in the two-way factorial mixed ANOVA for personal risk and a significant main effect of the independent variable in the independent *t*-test for societal risk, both specified in the analysis plan.
(2) There will be an interaction effect between future thinking and ease of imagination on perceived risk for specific climate-related risk events, so that the difference between EFT and SFT in perceived risk for specific climate-related risk events will be stronger for the difficult-to-imagine scenarios than for the easy-to-imagine scenarios. We predict this effect only for the measure of personal risk. Statistically, this will be reflected by a significant interaction effect in the two-way factorial mixed ANOVA for personal risk specified in the analysis plan.
(3) There will be an interaction effect between future thinking and form of risk perception, so that there will be a smaller difference between perceived personal risk and perceived societal risk for participants in the EFT-condition than in the semantic future thinking-condition. We predict this interaction for perceived risk for specific climate-related risk events. Statistically, this will be reflected by a significant interaction in the two-way mixed ANOVA specified in the analysis plan.
(4) There will be a main effect of future thinking on perceived risk of climate change in general, so that participants in the EFT-condition will perceive the risk of climate change in general as higher than participants in the SFT-condition. Statistically, this will be reflected by a significant main effect in the two independent *t*-tests for perceived personal risk of climate change in general and perceived societal risk for climate change in general specified in the analysis plan.
(5) There will be a main effect of future thinking on willingness to give monetary support to an environmental organization, so that participants assigned to the EFT-condition will be willing to donate a higher amount of money to this organization than participants assigned to the SFT-condition. Statistically, this will be reflected as a significant effect in the independent *t*-test specified in the analysis plan.

### Sample and Procedure

#### Sample Size Calculation

We used G^∗^Power 3 (Version 3.1.9.4) to conduct a power analysis to calculate sample size for the analyses testing the main hypotheses. To ensure that we had sufficient power for our most sensitive tests, we conducted power analyses based on the *post hoc* test we aimed to use to follow up the main analysis for hypothesis 2 and hypothesis 3 (independent *t*-test with an adjusted alpha-level). For the *post hoc* independent *t*-test following the main analysis testing our hypotheses, G^∗^Power suggested a minimal sample size of 128 (effect size *d* = 0.5, alpha level = 0.025, power = 0.80, allocation ratio 1). To compensate for a potential uneven number of participants in the conditions and participants dropping out of the study, we aimed to recruit a sample of minimum 170 participants.

#### Sample

170 participants (36 men, 133 women) were recruited from the Faculty of Psychology, University of Bergen. Participants were on average 22 years old (*SD* = 3.43), and had an average of 1.57 years of higher education (*SD* = 1.87).

#### Procedure

As per the pre-registration, participants were randomly assigned to conditions with simple randomization using the randomizer function in Qualtrics. This means that every participant entering the online survey link had an equal (25%) chance to be assigned to one of four blocks: (1) EFT with the easy scenario first and the difficult scenario last; (2) EFT with the difficult scenario first and the easy scenario last; (3) SFT with the easy scenario first and the difficult scenario last or (4) SFT with the difficult scenario first and the easy scenario last.

Participants arrived at the Faculty of Psychology and were informed that the study they were about to participate in focused on how people think about climate change. Thereafter, they were seated at individual desks. They read an introductory statement for the study and indicated their informed consent. Following this, they were asked to indicate their gender, age and how many years of higher education they had. Subsequently, the participants were exposed to the experimental induction.

In the EFT-condition (*n* = 85), participants were asked to imagine specific, personal episodes in the future. The two easy scenarios asked participants to imagine two personal scenarios where they are affected by an increase in the number of floods. Specifically, participants were asked to imagine experiencing flooded roads while in a car and experiencing flooding in a basement. The two difficult scenarios asked participants to imagine two personal scenarios where they are affected by an increase in extreme temperature. Specifically, participants were asked to imagine experiencing taking a bath in a lake with dangerous bacteria and experiencing a landslide as a result of an increase in extreme temperature.

Participants in the SFT-condition (*n* = 85) were asked to think about the future in an abstract, impersonal way. The two easy scenarios asked participants to think about how the frequency of floods may change in the future. The two difficult scenarios asked participants to think about how the frequency of extreme temperature may change in the future.

A full description of the wording used in the experimental manipulations can be found in the OSF-folder for this study.^2^ After the experimental manipulation, participants answered the manipulation checks, the questions measuring potential covariates and the perceived risk of the specific climate-related risk event. Thereafter, they answered questions pertaining to their perceived risk of climate change, before being given the opportunity to donate money to an environmental organization. Finally, they were given a written debrief describing the purposes of the study in full.

### Measures

#### Demographic Measures

Gender was measured with three categories (1 = male, 2 = female, 3 = do not wish to report gender). Age was measured with one item, *How old are you?* Years of education was measured with one item, *How many years of higher education have you completed?*

#### Manipulation Checks

In accordance with other studies on EFT, we included phenomenological measures to assess participants’ experience of their own thought content (Miloyan and McFarlane, 2019). Using phenomenological, self-report measures as manipulation checks on participants’ thinking resonates with the assumption that when assessing private mental content, participants may have better access to their own mental processes than any external observers (Miloyan and McFarlane, 2019; Miloyan et al., 2019).

An index of vividness was constructed from the following three 7-point Likert-type items, with endpoints 1 (completely disagree) and 7 (completely agree): *I imagined one specific event*, *my thoughts were vivid*, *my thoughts were concrete*. The scores on the index had excellent internal consistency (α = 0.84).

The following three constructs were measured with one item each, on a scale from 1 to 7, with endpoints 1 (completely disagree) and 7 (completely agree): Degree of future thinking (*I thought about the future*), autonoetic consciousness (*I was taken forward in time to when the events might take place*) and field perspective (*I experienced the events through my own eyes*).

#### Covariates

The following three constructs were measured with one item each, on a scale from 1 to 7, with endpoints 1 (completely disagree) and 7 (completely agree): Judged realism (*the imaginations of the events were realistic*), subjective difficulty of generating a scenario (*it was easy to perform the task where I was asked to imagine events*) and perceived temporal distance (*when you imagined the events, how far away in time did you perceive them to be?*). Time spent on imagining future episodes or thinking about future semantic events was measured in seconds using a timing question in Qualtrics.

#### Dependent Variable

Perceived risk was measured using two items for specific climate-related risk events and two items for climate change in general. Participants were asked to judge personal and societal risk for specific climate-related risk events, specifically for more floods after reading the flood scenarios in the two experimental conditions, and for more extreme temperature after reading the temperature-related scenarios in the two experimental conditions. At the end of the questionnaire, participants were asked to judge personal and societal risk for climate change in general.

Specifically, participants were asked the following questions:

(1) Specific climate-related risk events
  (a) How risky is it if you experience more floods/extreme temperature in the future?
  (b) How risky is it for people in Norway if they experience more floods/extreme temperature in the future?

(2) Climate change in general
  (a) How risky is it if you experience climate change in the future?
  (b) How risky is it for people in Norway if they experience climate change in the future?

All four items were on a scale from 1 to 7, with endpoints not risky (1) and very risky (7).

Willingness to give monetary support to an organization dealing with climate change issues was measured using one item asking participants how much of their compensation for participating in the experiment (50 NOK), if any, they would donate to Nature and Youth, a youth pro-environmental organization. Specifically, participants were informed that they would have the opportunity to donate any amount of the 50 NOK they received as compensation immediately after leaving the laboratory and were then asked to indicate whether they wished to donate 0, 10, 20, 30, 40, or 50 NOK to Nature and Youth. Alpha values mentioned in this materials and methods section were interpreted in relation to Ponterotto and Ruckdeschel (2007).

### Ethics Statement

This work complied with the general guidelines for research ethics by the Norwegian National Committees for Research Ethics in the Social Sciences and the Humanities (NESH). The data were not covered by the Norwegian Personal Data Act, making this project exempt from the need to submit a formal application to the Data Protection Official for Research. Furthermore, as our research questions were not related to health, we were not required to formally apply to the Regional Committee for Medical and Health Research Ethics, because the study was not covered by the Norwegian Health Research Act. Participants marked their informed consent before participation, in accordance with the Declaration of Helsinki (World Medical Association, 2013). The participants received a written debrief explaining the purposes of the study after completing the questionnaire.^2^

## Results

### Analysis Plan

All analyses were conducted using IBM SPSS [Version 25]. A cut-off criterion in the form of a *p*-value of 0.05 was used in the analyses. For the *t*-tests testing an effect of order of presentation, a Bonferroni correction to the alpha was applied (0.05/4 = 0.0125).

To test whether there was an order effect for the presentation of the scenarios in the within-subjects variable, four independent *t*-tests were conducted, with order of presentation (order 1 vs. order 2) as the between-subjects variable, and perceived personal risk of specific climate-related risk events, perceived societal risk of specific climate-related risk events, perceived personal risk of climate change in general and perceived societal risk of climate change in general as dependent variables.

To test the first hypothesis concerning the manipulation check items, three independent *t*-tests were conducted, with type of future thinking (episodic vs. semantic) as the independent variable, and vividness, autonoetic consciousness and field perspective as dependent variables.

To test the second hypothesis concerning the manipulation check items, two one-sample *t*-tests were conducted, comparing scores on the item measuring degree of future thinking to the scale midpoint (4) in both the EFT-condition and the SFT-condition. To test the third hypothesis concerning the manipulation check items, one dependent *t*-test was run with type of scenario (easy vs. difficult) as the independent variable, and perceived difficulty as the dependent variable.

For the main analyses testing hypothesis 1 and 2, we used a two-way factorial mixed ANOVA to test the main effect of future thinking (episodic vs. semantic) and the interaction effect between future thinking and type of scenario (easy vs. difficult) on personal risk. For the effect of future thinking on societal risk (main hypothesis 1), we used an independent *t*-test, with future thinking (episodic vs. semantic) as the independent variable and societal risk as the dependent variable. For the main analysis testing hypothesis 3, concerning the interaction between future thinking and form of perceived risk, we used a two-way mixed ANOVA, with future thinking (episodic vs. semantic) as the between-subjects variable and form of risk (personal risk vs. societal risk) as the within-subjects factor, and perceived risk as the dependent variable.

For the main analysis testing hypothesis 4, concerning the effect of future thinking on perceived risk for climate change in general, two independent *t*-tests were conducted, with future thinking (episodic vs. semantic) as the independent variable, and perceived personal risk of climate change in general and perceived societal risk of climate change in general as the dependent variables.

For the main analysis testing the effect of future thinking (EFT vs. SFT) on the measure of willingness to give monetary support to an environmental organization (hypothesis 5), one independent *t*-test was conducted, with future thinking (episodic vs. semantic) as the independent variable, and scores on the willingness to donate measure as the dependent variable.

To test whether any of the four measured potential covariates (amount of time spent on the exercise, judged realism, subjective difficulty of generating a scenario or perceived temporal distance) correlated with the dependent variable, four Pearson correlations were conducted for each of the dependent variables (perceived personal risk of specific climate-related risk events, perceived societal risk of specific climate-related risk events, perceived personal risk of climate change in general and perceived societal risk of climate change in general). In accordance with the pre-registration, the alpha-value was corrected, as specified in the beginning of this analysis plan section. For all significant associations with the dependent variable, we corrected for covariates in additional analyses, available in the OSF-folder.^2^

### Data Exclusion and Missing Values

To define outliers, we used the MAD-median rule (Wilcox, 2012). We used grouped data in the analyses, and as such, outliers were evaluated separately for each experimental condition (Tabachnick and Fidell, 2014). Unless otherwise reported, no outliers were detected for the variables. In the cases where outliers were present, these were retained in the analyses, and all the analyses involving scores with outliers were conducted both with and without outliers (Howitt and Cramer, 2011). The additional analyses without outliers are available in the OSF-folder^2^, and any differences in results are noted. As the number of missing cases was below 5%, we excluded cases analysis-by-analysis in SPSS (Tabachnick and Fidell, 2014). This was specified in our pre-registration.

### Main Results

#### Preliminary Analyses

Conditions were comparable on demographic variables. The distribution of gender was not significantly different depending on condition, χ^2^ (2, *N* = 170) = 1.01, *p* = 0.60, φ = 0.07. The scores on the higher education variable were not significantly different depending on condition, *t*(168) = −0.70, *p* = 0.49, *d* = −0.11. This did not change when outliers were excluded, *t*(152) = −0.17, *p* = 0.87, *d* = −0.02.

#### Order Effects

There was no order effect on the measure of perceived societal risk for specific climate-related risk events, perceived personal risk for climate change in general or perceived societal risk for climate change in general. Table 1 gives an overview of the tests for the order effects. There was, however, an order effect on the measure of perceived personal risk for specific climate-related risk events: Participants who were presented with extreme temperatures before flooding (*M* = 5.68, *SD* = 1.05) perceived the risk of specific climate-related risk events as higher than participants who were presented with flooding before extreme temperatures (*M* = 5.29, *SD* = 1.09), *t*(168) = −2.41, *p* = 0.02, 95% CI [−0.72, −0.07], *d* = 0.39. However, as per the pre-registration, we used a Bonferroni adjustment on the α-value, and the order effect was not significant with the new threshold (0.05/4 = 0.0125). Analyses controlling for the order effect are still available in the OSF-folder.^2^ Unless otherwise noted, the order effect did not change or interact with any other main effects or interaction effects.

**TABLE 1:** Independent *t*-tests comparing scores on perceived risk measures for order of presentation.

	*t*	*df*	*p*	*d*	95% CI
Specific perceived personal risk	−2.41	168	0.02	0.39	[−0.72, −0.07]
Specific perceived societal risk	−0.70	168	0.48	−0.11	[−0.40, 0.19]
General perceived personal risk	0.44	168	0.66	0.07	[−0.20, 0.32]
General perceived societal risk	0.20	168	0.84	0.02	[−0.23, 0.29]

#### Manipulation Checks

As predicted, there was a significant difference on the index of vividness depending on condition, *t*(166) = 2.30, *p* = 0.02, 95% CI [0.05, 0.63], *d* = 0.35. Participants in the EFT-condition (*M* = 5.51, *SD* = 0.97) reported a significantly higher degree of vividness than participants in the SFT-condition (*M* = 5.17, *SD* = 0.96).

Contrary to predictions, there was no significant difference on the measure of autonoetic consciousness depending on condition, *t*(166) = 1.10, *p* = 0.27, 95% CI [−0.18, 0.66], *d* = 0.17. Participants in the EFT-condition (*M* = 4.91, *SD* = 1.47) did not report a significantly greater degree of autonoetic consciousness than participants in the SFT-condition (*M* = 4.67, *SD* = 1.30). As predicted, there was a significant difference in field perspective depending on condition. As Levene’s test for equality of variances was significant (*F* = 6.55, *p* = 0.01), indicating that the variances in the conditions were unequal, a t-value with corrected degrees of freedom was used. Participants in the EFT-condition (*M* = 5.88, *SD* = 1.12) reported a significantly higher degree of field perspective than participants in the SFT-condition (*M* = 4.24, *SD* = 1.50), *t*(155.50) = 8.14, *p* < 0.001, 95% CI [1.25, 2.05], *d* = 1.24.

Two one-sample *t*-tests were conducted to explore the degree of future thinking in the two experimental conditions. The *t*-test for the EFT-condition (*t*(84) = 7.17, *p* < 0.001, 95% CI [0.91, 1.60], *d* = 0.78) and the *t*-test for the SFT-condition (*t*(84) = 16.85, *p* < 0.001, 95% CI [1.77, 2.24], *d* = 1.83) were significant. Participants in the EFT-condition (*M* = 5.26, *SD* = 1.61) and the SFT-condition (*M* = 6.01, *SD* = 1.10) had higher scores than the midpoint of 4, suggesting that participants were engaged in future thinking in both experimental conditions.

Contrary to expectations, there was no significant difference in how difficult participants perceived imagining floods and imagining extreme temperature to be, *t*(169) = −0.50, *p* = 0.96, 95% CI [−0.24, 0.23], *d* = 0.17. Difficulty in imagining floods (*M* = 4.93, *SD* = 1.73) was not rated as greater than difficulty in imagining extreme temperature (*M* = 4.93, *SD* = 1.66).

#### Covariates

Both judged realism and perceived temporal distance were significantly associated with perceived personal risk for specific climate-related risk events, perceived societal risk for specific climate-related risk events and perceived personal risk for climate change in general. Table 2 gives an overview of the covariate analyses. Higher judged realism of the imagined events, and reduced perceived temporal distance, was associated with a higher degree of perceived risk. Judged realism, but not perceived temporal distance, was significantly associated with perceived societal risk for climate change in general, with a higher judged realism being associated with a higher degree of perceived risk (Table 2). As such, additional analyses involving the covariates were conducted for the main analyses, available in the OSF-folder.^2^ As a general note, results were comparable.

**TABLE 2:** Pearson correlations between risk perception and covariates.

	Judged realism (*N* = 170)	Subjective difficulty of generating a scenario (*N* = 170)	Perceived temporal distance (*N* = 170)	Amount of time spent on the task (*N* = 169)
Personal risk	0.31 (*p* < 0.01)	0.18 (*p* < 0.05)	−0.30 (*p* < 0.01)	0.06 (*p* = 0.44)
Societal risk	0.28 (*p* < 0.01)	0.06 (*p* = 0.44)	−0.25(*p* < 0.01)	0.13 (*p* = 0.08)
General personal risk	0.33 (*p* < 0.01)	0.09 (*p* = 0.26)	−0.19 (*p* = 0.01)	0.05 (*p* = 0.50)
General societal risk	0.36 (*p* < 0.01)	0.01 (*p* = 0.86)	−0.15 (*p* = 0.05)	0.10 (*p* = 0.22)

#### Effect of Episodic Future Thinking and Type of Scenario (Easy vs. Difficult to Imagine) on Perceived Risk for Specific Climate-Related Risk Events

**FIGURE 1:**
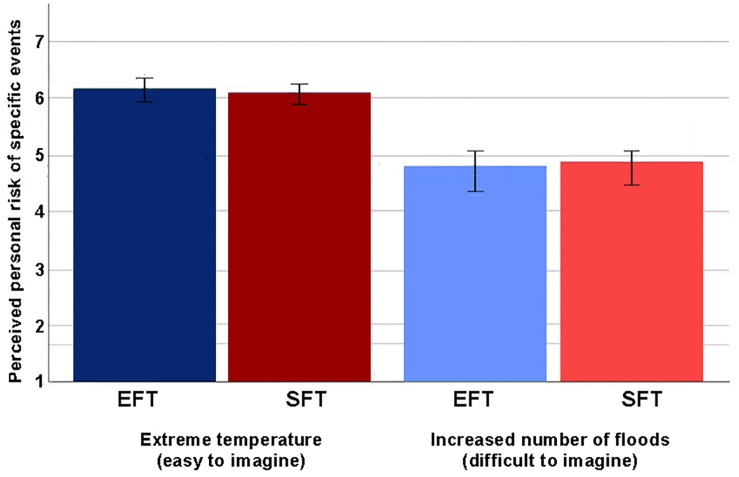
Interaction between future thinking and type of scenario on the perceived risk of specific events.

**FIGURE 2:**
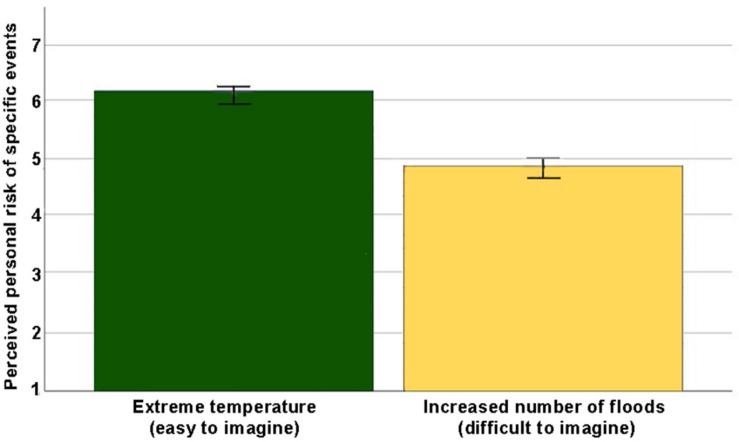
Main effect of type of scenario on the perceived risk of specific events.

To explore whether there was an interaction between form of future thinking and type of scenario on perceived personal risk for specific climate-related risk events, we conducted a two-way factorial mixed ANOVA. There was no significant interaction between future thinking and type of scenario, Wilks’ Lambda = 0.99, *F*(1,168) = 1.17, *p* = 0.28, η_p__2_ = 0.01. Figure 1 shows the mean perceived risk for each type of scenario, divided by type of future thinking. There was a significant main effect of type of scenario, Wilks’ Lambda = 0.54, *F*(1,168) = 141.96, *p* < 0.001, η_p__2_ = 0.46. Figure 2 shows the main effect of type of scenario on perceived personal risk for specific climate-related risk events. Participants perceived the personal risk of extreme temperature (*M* = 6.12, *SD* = 1.04) as higher than the personal risk of floods (*M* = 4.82, *SD* = 1.52). This did not change when controlling for outliers or when including significant covariates.

**FIGURE 3:**
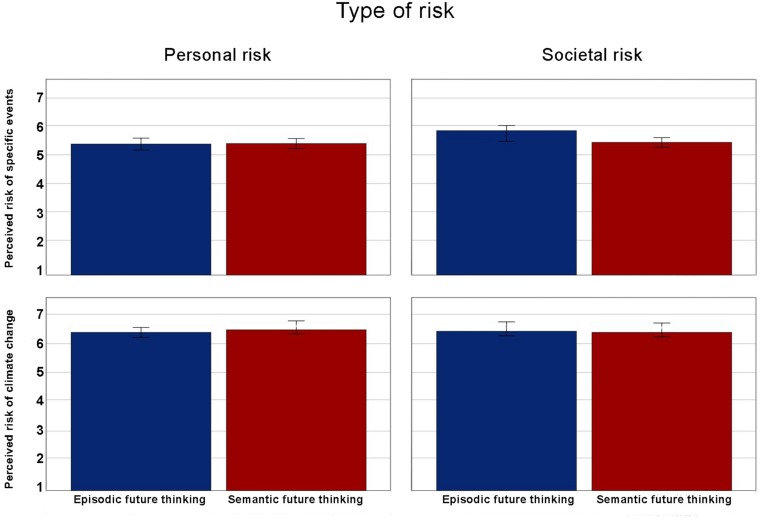
Main effect of future thinking on perceived risk measures.

There was no significant main effect of future thinking, *F*(1,168) = 0.05, *p* = 0.89, η_p__2_ = 0.00. Figure 3 shows the main effect of future thinking on all perceived risk measures included in the study. Table 3 shows the perceived risk of climate change, extreme temperature and floods for both episodic and semantic future thinking, and for both perceived personal risk and perceived societal risk.

**TABLE 3:** Overview of perceived risk scores on different outcome measures for the different conditions.

	Episodic future thinking				Semantic future thinking			
	Personal risk		Societal risk		Personal risk		Societal risk	
	*M*	*SD*	*M*	*SD*	*M*	*SD*	*M*	*SD*
Flooding (difficult to imagine)	4.75	1.65	5.60	1.25	4.89	1.38	5.20	1.30
Extreme temperature (easy to imagine)	6.16	1.08	5.98	1.10	6.07	1.00	5.86	1.01
Climate change	6.46	0.93	6.51	0.85	6.56	0.76	6.46	0.85

*Displays means on perceived personal and societal risk for specific events and for climate change in general.*

There was also a significant interaction between type of scenario and order of presentation, Wilks’ Lambda = 0.97, *F*(1,166) = 5.26, *p* = 0.02, $\eta_{\text{p}}^{2}$ = 0.03. Participants presented with extreme temperature prior to floods (*M* = 5.16, *SD* = 1.42) perceived the risk of floods as significantly higher than participants presented with floods prior to extreme temperature (*M* = 4.53, *SD* = 1.54). In other words, while participants generally perceived the risk of extreme temperatures as greater than the risk of floods, the perceived risk of floods depended on whether participants were presented with extreme temperature or floods as scenarios first.

To explore the effect of future thinking on societal risk, we conducted an independent *t*-test. Contrary to expectations, there was no main effect of future thinking on perceived societal risk for specific climate-related risk events, *t*(168) = 1.74, *p* = 0.08, 95% CI [−0.03, 0.55], *d* = 0.27 (Figure 2). Participants in the EFT-condition (*M* = 5.79, *SD* = 0.98) did not report a significantly greater degree of societal risk for specific climate-related risk events than participants in the SFT-condition (*M* = 5.53, *SD* = 0.96). This did not change when controlling for outliers or when including significant covariates.

#### Effect of Episodic Future Thinking and Form of Risk (Personal vs. Societal) on Perceived Risk for Climate-Related Risk Events

To explore whether there was an interaction between form of future thinking and form of risk, we conducted a two-way factorial mixed ANOVA. There was a significant interaction between future thinking and form of risk, Wilks’ Lambda = 0.98, *F*(1,168) = 3.94, *p* = 0.05, $\eta_{\text{p}}^{2}$ = 0.02. However, as demonstrated by prior tests, there was no significant difference in either personal or societal risk depending on future thinking. As the tests for both personal and societal risk have already been conducted as main analyses, we deviated from the pre-registration by not conducting these after finding the significant interaction. The interaction effect between future thinking and form of risk on perceived risk for climate-related risk events was qualified by a significant interaction between form of risk and order of presentation, Wilks’ Lambda = 0.97, *F*(1,166) = 4.78, *p* = 0.03, $\eta_{\text{p}}^{2}$ = 0.03. The difference between perceived personal risk (*M* = 5.29, *SD* = 1.09) and perceived societal risk (*M* = 5.61, *SD* = 0.98) for participants presented with floods before extreme temperature was greater than the difference between perceived personal risk (*M* = 5.68, *SD* = 1.05) and perceived societal risk (*M* = 5.72, *SD* = 0.98) for participants presented with extreme temperature before floods.

#### Effect of Episodic Future Thinking on Perceived Risk for Climate Change in General

To test whether future thinking impacted perceived personal and societal risk of climate change in general, we conducted two independent *t*-tests. Contrary to predictions, there was no significant effect of future thinking on perceived personal risk of climate change, *t*(168) = −0.81, *p* = 0.42, 95% CI [−0.36, 0.15], *d* = −0.12 (Figure 2). Participants in the EFT-condition (*M* = 6.46, *SD* = 0.93) did not perceive the personal risk of climate change as greater than participants in the SFT-condition (*M* = 6.56, *SD* = 0.76). Contrary to predictions, there was no significant effect of future thinking on perceived societal risk of climate change, *t*(168) = 0.36, *p* = 0.72, 95% CI [−0.21, 0.31], *d* = 0.06 (Figure 2). Participants in the EFT-condition (*M* = 6.51, *SD* = 0.85) did not perceive the societal risk of climate change as greater than participants in the SFT-condition (*M* = 6.46, *SD* = 0.85).

#### Effect of Episodic Future Thinking on Willingness to Donate

Summing up the intended donation amounts, participants were willing to donate a total amount of 2870 NOK (approximately 1/3 of the received compensations). The total amount of donated money was 2390 NOK. This suggests that the intended donation measure may have overestimated the total amount of money participants were willing to donate.

To test whether future thinking impacted willingness to donate, we conducted an independent *t*-test. Contrary to predictions, there was no effect of future thinking on willingness to donate, *t*(168) = −1.95, *p* = 0.05, 95% CI [−12.07, 0.07], *d* = −0.30. Participants in the EFT-condition (*M* = 13.89, *SD* = 19.58) were not more willing to donate money than participants in the SFT-condition (*M* = 19.88, *SD* = 20.50). Removing the outliers, there was a significant effect of future thinking on willingness to donate. As Levene’s test for equality of variances was significant (*F* = 24.02, *p* < 0.001), indicating that the variances in the conditions were unequal, a *t*-value with corrected degrees of freedom was used. Participants in the EFT-condition (*M* = 13.89, *SD* = 19.58) were more willing to donate money than participants in the SFT-condition (*M* = 8.71, *SD* = 10.48), *t*(134.37) = 2.06, *p* = 0.04, 95% CI [−0.22, 10.13], *d* = 0.32. However, as per the pre-registration, the solution with outliers included was kept, and as such, we conclude that participants did not donate differently according to experimental condition.

## Discussion

### Summary

Based on prior research suggesting an effect of episodic future thinking (EFT) on perceived risk, we conducted an experiment to test whether EFT would increase the perceived risk of climate-related risk events, compared to semantic future thinking (SFT). Additionally, pursuing an explanation for prior findings, we wished to test whether high imaginability of consequences would moderate this effect, and decrease the effect of EFT on perceived risk. We hypothesized that there would be a main effect of EFT for both perceived personal and societal risk of specific climate-related risk events, and also an interaction between EFT and imaginability for perceived personal risk. Additionally, we predicted an interaction between EFT and form of risk perception, so that the difference between personal and societal risk for specific climate-related risk events would be lower in the EFT-condition than in the SFT-condition. As the results show, we found no support for any main effect of EFT, any interaction with imaginability, or any interaction with form of risk perception.

Additionally, we predicted a main effect of EFT on perceived personal and societal risk of climate change in general, and an effect on intentions to donate money to a pro-environmental organization. We found no support for these hypotheses either. To summarize, there was no support for an effect of EFT on perceived risk or behavioral intentions, and no support for any interaction with either imaginability or form of risk perception. Contrary to our hypotheses and most prior research, but consistent with Bø and Wolff (2019), the results do not support an effect of EFT on perceived risk, and do not suggest any moderation of this effect by the imaginability of a risk event.

Notwithstanding the lack of evidence for an effect of EFT on perceived risk, results on the phenomenological manipulation check measures suggest that participants engaged in the correct form of future thinking according to condition. Specifically, participants in both conditions reported that their thinking was directed toward the future, and participants in the EFT-condition reported that their thoughts were more vivid and that they had a greater degree of field perspective than participants in the SFT-condition. As both vividness and field perspective are integral to EFT, the results suggest that our experimental manipulations were successful. An important exception is that participants in the EFT-condition did not report a greater degree of autonoetic consciousness than participants in the SFT-condition. This could be both due to the fact that participants in the EFT-condition did not experience the event with autonoetic consciousness, or that participants in both conditions did. This may imply an unsuccessful experimental manipulation, as autonoetic consciousness is considered a principal element in EFT (Tulving, 1985; McCarroll, 2019). Despite this exception, the results generally suggest that our experimental manipulations were successful, in spite of no differences in perceived risk between conditions.

Given a functioning experimental manipulation, no effect of EFT on perceived risk suggests that future thinking may not be important for perceived risk. However, prior research suggests that vividness, personal relevance and perceived psychological proximity, which are all associated with EFT, predict perceived risk (Slovic et al., 1981; Zwickle and Wilson, 2013; McDonald et al., 2015; van der Linden, 2015; Bulley et al., 2016). Additionally, episodic thoughts can count as vicarious evidence similarly to personal experience with extreme weather events, and studies suggest that personal experience increases climate change risk perceptions (Weber, 2006, 2016; McDonald et al., 2015). Hence, our null finding runs counter to results from prior research. There are several possible explanations for these results, which are important to consider in order to contextualize the association between future thinking and perceived risk, and to identify directions for future, fruitful research.

### Potential Explanations

#### Conceptual Explanations

If, as we assumed, psychological distance is a relevant mechanism for EFT, one potential explanation is that bringing climate change closer does not necessarily strengthen climate change risk perceptions. In the context of psychological distance and perceptions of climate change, some authors have argued that an increased psychological distance may be beneficial, for example by being a prerequisite to some complex emotional reactions to climate change, such as guilt or shame, which motivate pro-environmental behavior (Ejelöv et al., 2018). Also, others have argued that not all aspects of psychological distance matter in explaining climate change perceptions (Jones et al., 2017). It is plausible that EFT may impact some forms of psychological distance that are not necessarily relevant for climate change risk perceptions. If EFT serves to bring climate change closer, but this closeness is insufficient to heighten risk perceptions, then EFT may not be important in explaining climate change risk perceptions.

A related, but opposed, explanation is that EFT may not have influenced psychological distance, and therefore not influenced climate change risk perceptions. If changes in psychological distance are responsible for effects of EFT on risk perceptions, then a lack of difference in psychological distance between our experimental conditions may be a viable explanation. Supporting this explanation, a supplementary analysis indicated no significant difference between the EFT- and SFT-conditions in psychological distance^3^. Indeed, the means suggest that participants in the SFT-condition (*M* = 3.18, *SD* = 1.08) perceived the climate-related risk events as closer than participants in the EFT-condition (*M* = 3.55, *SD* = 1.43). However, as we also identified other mechanisms that could explain an effect of EFT, including vividness and vicarious evidence, and because we have no clear reasons to doubt these proposed mechanisms, a lack of difference in psychological distance should not alone be able to account for the lack of any effect of EFT on perceived risk.

#### Methodological and Statistical Explanations

There are also methodological explanations to our null findings, particularly centered around the effectiveness of the manipulation checks and the manipulations. As previously mentioned, there was no significant difference in autonoetic consciousness between the EFT- and SFT-conditions, despite this being a crucial characteristic of episodic thought (Tulving, 1985; McCarroll, 2019). While this may suggest that our experimental manipulation was ineffective, the other manipulation checks support the effectiveness of the manipulation, and the mean difference for the scores on autonoetic consciousness was in the predicted direction.

One objection to our argument that the manipulation was effective may be that while the answers from participants suggested that they engaged in either EFT or SFT, demand characteristics could explain these answers. Specifically, assuming that the participants wished to be “good subjects,” it is possible that they answered according to what they understood they were being asked to do, instead of according to what form of thinking they were engaged in (Orne, 1962). This would explain the lack of an experimental effect, in spite of a seemingly successful experimental manipulation. However, if participants acted in accordance with demand characteristics, they should also have followed the instructions, which means that they should also have actually engaged in either EFT or SFT. In other words, participants would either genuinely engage in EFT or SFT and answer the manipulation check measures correctly, or not engage in EFT or SFT and not answer the manipulation check measures correctly. While demand characteristics may seem an alluring explanation, they cannot account for the discrepancy between a successful manipulation and the lack of an experimental effect.

Methodological explanations may also be applied in an attempt to understand why we found no interaction effect between EFT and imaginability. One explanation is that ease of imagination was not successfully manipulated. The results from our pilot study gave good grounds for assuming that it would be more difficult to imagine flooding than extreme temperature. Nonetheless, in the main study, there were no differences in judged difficulty of imagining the scenario depending on type of scenario. This suggests that we may have failed to select climate-related risk events which were sufficiently different in imaginability. While this may explain the lack of an interaction effect, it does not explain the lack of a main effect of EFT on perceived risk.

Finally, a self-evident statistical explanation to why we found no effect of EFT is that the effect of EFT is too small to detect with our number of participants. We based our *a priori* power analysis on prior studies finding medium to large effects of EFT, particularly on decision making, but also for risk perception (Lee et al., 2018; Hollis-Hansen et al., 2019). Nonetheless, these may be overestimations of the effect, owing to the unintentional use of questionable research practices or publication bias favoring significant effects (Ferguson and Heene, 2012; Wicherts et al., 2016). Similar explanations have been used to explain why effect sizes found in replication attempts are typically smaller than in the original studies (Open Science Collaboration, 2015; Camerer et al., 2018). If the effect size is smaller than previously assumed, a lack of statistical power may explain why we found no effect of EFT. However, given prior research on EFT, we argue that it is improbable that the effect is too small to detect in our experimental design, as shown in the *a priori* power analysis.

### Additional Methodological Considerations

While the following considerations are peripheral in explaining why we found no effect of EFT on perceived risk, they still affect our interpretation of the results. One example is the measurement of risk perception, which was operationalized using general measures, reflecting the importance of both perceived probability and perceived severity (Brun, 1994). These measures are also consistent with the original study on the effect of EFT on the perceived risk of terrorism and were thus suitable to follow up explanations for the results in that study (Bø and Wolff, 2019). If EFT primarily increases the perceived probability of the consequences, but not the perceived severity of the consequences, this may explain why there were no effects in our study, because perceived risk is strongly influenced by probability neglect. This may also explain why EFT increased perceived risk in a prior study which used a measure emphasizing perceived probability (Lee et al., 2018). Additionally, multidimensional measures of risk perception may be more suitable to reflect the construct, particularly in the context of climate change (van der Linden, 2015; Wilson et al., 2019). If there is an effect of EFT on climate change risk perception, but our measure did not accurately reflect the construct, this may explain our null finding. Exploring whether there is an effect using a multidimensional measure of perceived risk is an important avenue in future research.

Another methodological consideration concerns our measure of intentions to donate money to a pro-environmental organization. To gain an understanding of how future thinking could potentially impact behavior, we included an intention measure. As shown by the results, there was a discrepancy between the amount participants were willing to donate (2870 NOK) and the actual amount donated (2390 NOK). This suggests that our measure may not have been a good proxy for behavior, despite there being theoretical reasons to assume a strong relationship between specific behavioral intentions and behavior (Ajzen, 2001). While other measures may be relevant to include in future research, we chose our measure to ensure the anonymity of our research participants, as the context precluded recording actual behavior without breaching anonymity.

An important methodological concern is the sample used in the experiment for the main study, which only consisted of psychology students. This is a very homogenous population, which may be assumed to have above-average attention to environmental risks, thus limiting the generalizability of the findings. Additionally, this sample of students may have already been sufficiently proficient in imagining both personal consequences of floods and extreme temperature prior to being included in the study, potentially explaining why we were not able to successfully manipulate the imaginability of the climate-related risk events. Although we have good reason to believe that our manipulation of future thinking was successful, which helps build confidence in our conclusions regarding the experimental effect (or lack thereof), our choice of sample makes it necessary to conduct further research with more ecologically valid samples. Extending this line of research with different samples thus represents an important aim for future studies.

One closing methodological issue is the inequality in the gender distribution, with women accounting for approximately 78 percent of the sample. With prior research suggesting gender differences in some areas of risk perception, we might get different results with a more equally distributed sample (Hicks and Brown, 2013). However, there are no clear arguments for assuming that gender moderates either the relationship between EFT and perceived risk or the interaction between EFT and imaginability. Moreover, the gender balance amongst psychology students, the population from which we sampled, is unequal in favor of women (Database for Statistics on Higher Education, 2019). Based on this, we argue that the inequality in the sample does not pose a credible threat to the validity of our results.

### Future Research and Implications

The results have important ramifications for understanding the relationship between EFT and perceived risk, and potentially also for understanding the relationship between EFT and decision making. Regarding perceived risk, the effect of EFT on climate change risk perception may be more nuanced than previous research would suggest (Lee et al., 2018). More generally, there does not seem to be support for an effect of EFT for either climate change risk perceptions or terror risk perception (Bø and Wolff, 2019). Testing both hazards which are easy to imagine initially (terrorism), and climate-related risk scenarios that are either easy to imagine initially or difficult to imagine initially demonstrated no effects. However, we cannot exclude the possibility of an effect in future studies with more diverse samples than the ones we included in our studies, and future research needs to address both the limitation in ecological validity and the possibility of an effect for different hazards than terrorism and climate change.

While EFT does not seem important in explaining climate change risk perceptions, it may still matter for other climate change perceptions. For example, some distinguish between different forms of climate change knowledge, such as knowledge about consequences of climate change and procedural knowledge about how to respond to climate change (van der Linden, 2015). It may be that imagining personal, future episodes allows people to better understand the consequences of climate change or prepare them for how to respond to consequences of climate change. Exploring whether this is the case is an important question in future research on future thinking and climate change perceptions.

Regarding decision making, earlier studies suggest that EFT is important in intertemporal decision-making (Daniel et al., 2015; Bulley et al., 2016; Stein et al., 2018). It was plausible to assume that perceived risk may be an important mechanism in explaining this relationship. No association between EFT and perceived risk suggest that there may be other, more relevant mechanisms, such as psychological distance, which some studies show (Daniel et al., 2015). Exploring reasons for why future thinking impacts our decisions remains an important avenue for future research, particularly because people often engage in EFT in their daily lives (D’Argembeau et al., 2011; Barsics et al., 2016).

Reflecting the focus on EFT and decision making, future studies could explore whether EFT matters for decisions which are closely connected with risk perception. For terrorism, for example, one could imagine that engaging in EFT would make people gather additional information about their destination prior to traveling, implying an effect on decision making without any effect on risk perception. For climate risk, imagining specific episodes of experiencing risk events in the future may lead to more pro-environmental behavior, without such an association being explained by risk perception as a mechanism.

A final venue for future research could be to find another alternative to mental simulation as a substitute for lacking personal experience with the consequences of climate change. As emphasized in the introduction, one of the barriers to climate change engagement is the lack of personal experience with its consequences (McDonald et al., 2015). An alternative to using mental simulation as a substitute could be to use virtual reality-simulations of climate change consequences. This has been used in improving learning about climate change and could potentially be used as a tool to heighten risk perceptions and climate change engagement (Markowitz et al., 2018).

## Conclusion

How people perceive the risk of climate change affects their tendency to engage in pro-environmental behavior, meaning that understanding the precursors of climate change risk perception is of paramount importance. This pre-registered experiment tested EFT as a precursor to the perceived risk of specific risk events and climate change in general, and whether this effect would be moderated by imaginability of the risk event. Hypotheses were based on prior research suggesting that EFT may impact risk perception through vividness, perceived psychological proximity, personal relevance and vicarious evidence. Contrary to predictions, participants engaging in EFT did not perceive the risk as higher than participants engaging in SFT. The results suggest that EFT may not be important in explaining climate change risk perceptions. Still, whether EFT affects risk perceptions in other populations, can directly impact other climate change perceptions, or even directly impact pro-environmental behavior, remains a pressing question.

## Data Availability Statement

All datasets generated for this study are included in the article.

## Ethics Statement

Ethical review and approval was not required for the study on human participants in accordance with the local legislation and institutional requirements. The patients/participants provided their written informed consent to participate in this study.

## Author Contributions

SB and KW conceived of the idea, worked on the research design, critically revised the initial draft of the manuscript, and approved the final version prior to submission. SB gathered the data, conducted the analyses, and wrote the first draft of the manuscript.

## Conflict of Interest

The authors declare that the research was conducted in the absence of any commercial or financial relationships that could be construed as a potential conflict of interest.

## Footnotes

1 https://osf.io/g39bk

2 https://osf.io/h3vqb/

3 *t*(168) = 1.91, *p* = 0.06, 95% CI [−0.01, 0.75], *d* = 0.29.
